# Efficacy of Surgical Masks Versus N95 Respirators for the Prevention of COVID-19 in Dental Settings: A Systematic Review

**DOI:** 10.7759/cureus.37631

**Published:** 2023-04-16

**Authors:** Ali Alkhalaf, Essa Aljaroudi, Mohammed Al-Hulami, Balgis Gaffar, Khalid Almas

**Affiliations:** 1 Dentistry, College of Dentistry, Imam Abdulrahman bin Faisal University, Dammam, SAU; 2 Preventive Dental Sciences, College of Dentistry, Imam Abdulrahman bin Faisal University, Dammam, SAU

**Keywords:** dentistry, coronavirus, aerosols, n95 respirator, surgical mask, masks, covid-19

## Abstract

Coronavirus disease 2019 (COVID-19) is an infectious disease caused by severe acute respiratory syndrome coronavirus 2. (SARS-CoV-2). It spreads mainly through saliva droplets or nasal discharge. Dentists are among the professionals with the greatest risk of contracting and transmitting COVID-19. We compared the efficacy of surgical masks versus N95 respirators in preventing COVID-19 infection in dental settings. PubMed, Scopus, Web of Science, and Cochrane Library databases were searched. Search terms corresponded to a predefined PICOS (patient/population, intervention, comparison, and outcomes) question. The risk of bias was evaluated using AMSTAR-2 (A Measurement Tool to Assess Systematic Reviews-2), ROBIS (Risk of Bias in Systematic Reviews), and Health Evidence tools. A total of 191 articles were screened, and nine of them were further evaluated for eligibility, of which five articles (fulfilled the selection criteria) and were included in this study. Two studies concluded that surgical masks could provide equivalent protection to N95 respirators. Another study found that N95 respirators were superior to surgical masks. The fourth study found that better protection can be achieved when using surgical masks by the aerosol source than when the recipient uses an N95 respirator, while the last study concluded that surgical masks or N95 respirators alone do not provide full protection. Thus, according to this systematic review, N95 respirators provide better protection against COVID-19 infection compared to surgical masks.

## Introduction and background

Coronavirus disease 2019 (COVID-19) is an infectious disease discovered in Wuhan, Hubei Province, China in late December 2019 [1,2]. It spreads primarily through nasal discharge or saliva droplets from an infected person [1]. People are affected differently by COVID-19, the majority experience mild to moderate sickness and recover without hospitalization [1]. Healthcare workers (HCWs) caring for COVID-19-infected patients are most at risk, notoriously when operating aerosol-generating procedures [3]. Dentists are among the professionals at risk for contracting and transmitting COVID-19 owing the exposure to saliva, which is considered a reservoir for both symptomatic and asymptomatic infected patients [4]. Viral particles can be aerosolized in the dental environment by high-speed handpieces, ultrasonic scalers, three-way syringes, and other devices [5].

The airway is considered the main transmission route of COVID-19, accordingly, respiratory protective equipment (RPE) is an important tool for minimizing the transmission of COVID-19 infection [6]. RPE includes surgical masks and N95 respirators; surgical masks are disposable protective items that fit loosely over the wearer's mouth and nose to physically separate it from potential pathogens in the environment [7]. N95 respirators are designed to provide a very tight fit on the face and high filtration of airborne particles [7]. Gloves, gowns, face shields, and goggles are other items of essential personal protective equipment (PPE) [8]. HCWs have used surgical masks to avoid hand-to-face contact and stop the spread of respiratory droplets, although they may not be reliable in preventing aerosols [9]. On the other hand, the N95 respirators are meant to prevent the inhalation of aerosols when treating patients with suspected respiratory viral infections [6]. The use of N95 respirators by HCWs was highly recommended during the COVID-19 pandemic, but the available evidence is still controversial. Also, the shortage of PPE has made it difficult to apply adequate protection to HCWs, especially given the shortage of N95 respirators [3,10]. 

This systematic review was guided by the research question: Does the surgical mask provide similar protection from COVID-19 infection as the N95 mask? This systematic review aimed to compare the efficacy of surgical masks versus N95 respirators as a part of PPE in the era of the pandemic COVID-19 infection in dental settings.

## Review

Methods

We have adhered to the Preferred Reporting Items for Systematic Reviews and Meta-analyses (PRISMA) guidelines for reporting this review [11].

Search Terms and Eligibility Criteria

The systematic review included studies that met the predefined PICOs (patient/population, intervention, comparison, and outcomes). Inclusion criteria were applied as such: population (dentists, dental practice, dentistry), intervention (surgical masks, medical masks) comparison (N95 respirators), outcome (prevention of COVID-19/coronavirus/severe acute respiratory syndrome coronavirus 2. (SARS-CoV-2) infection/transmission), study design (randomized clinical trials, systematic reviews, meta-analysis, laboratory studies). We excluded studies evaluating other respiratory illnesses like influenza, flu, and common cold, studies not comparing surgical masks and N95 respirators, articles not written in English OR not available in the English language, as well as abstracts, surveys, grey literature, reviews (except for systematics reviews that evaluated both masks and N95), editorials, and comments.

Electronic Data Search and Study Selection

Two reviewers independently performed an electronic data search with a single search string developed using the predefined PICOS. Four electronic databases, PubMed, Scopus, Web of Science, and Cochrane Library, were searched for studies published from January 2020 to December 2021. The references were managed with Reference Manager 2.63.0 (2022; Mendeley Ltd, London, United Kingdom). 

**Figure 1:**
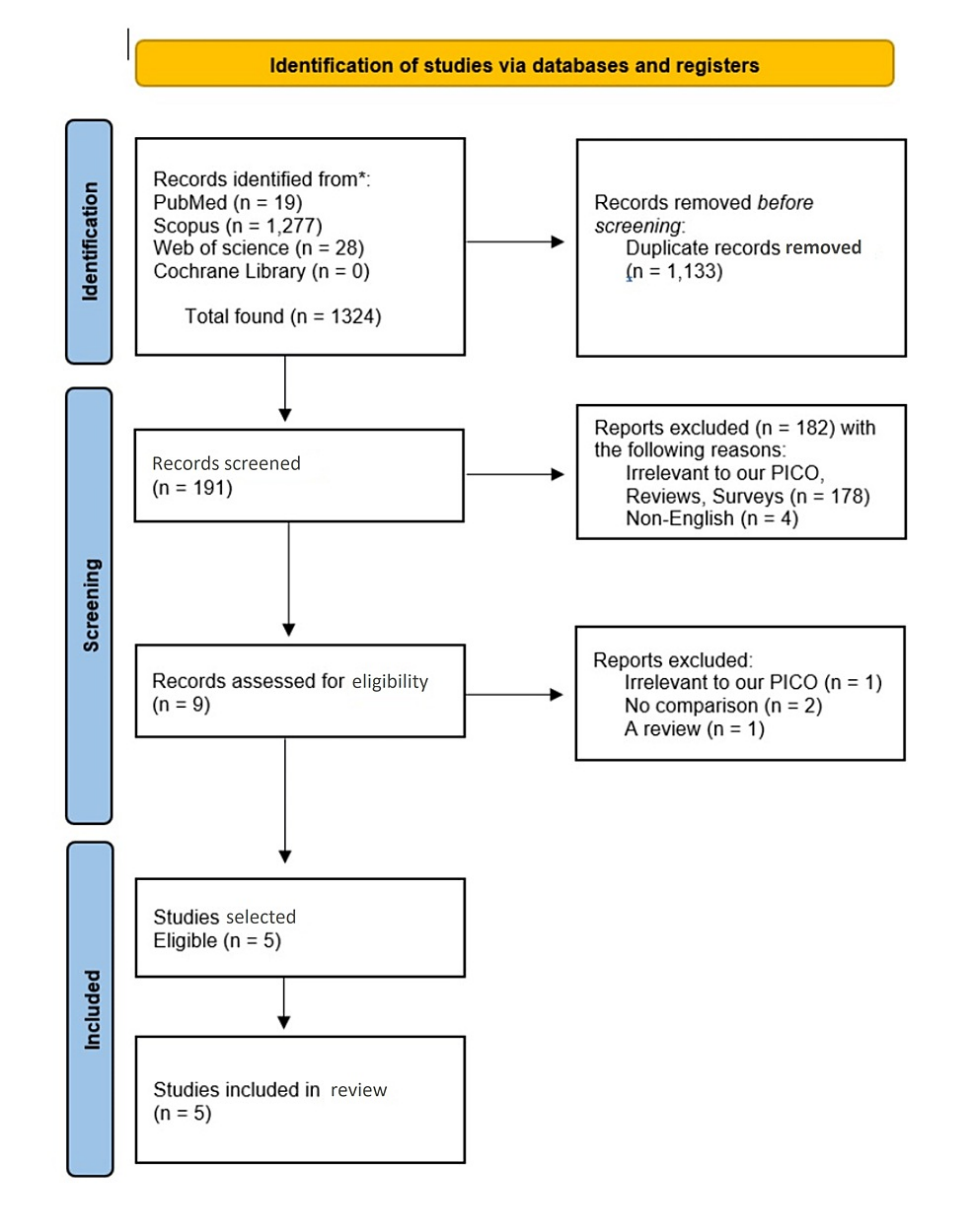
PRISMA flow chart of the study selection and literature search. PRISMA: Preferred Reporting Items for Systematic Reviews and Meta-analyses

The selection of articles was carried out in three stages: title and abstract screening in stage one, full-text review in stage two, and extraction and evaluation of the data in stage three. Two investigators independently performed each stage and a third reviewer was referred in case of any disagreement. Abstracts that did not contain the required information were included in the full-text analysis to avoid the exclusion of potentially relevant studies. The selected studies were read thoroughly. The PRISMA flowchart of the process is given in Figure 1.

Risk of Bias and Quality Assessment of Individual Studies

The risk of bias and quality assessment of the eligible studies was performed independently by two investigators. A third reviewer was consulted in case of any disagreement. The risk of bias of the included systematic reviews and meta-analysis was performed using three tools: AMSTAR-2 (A Measurement Tool to Assess Systematic Reviews), ROBIS (Risk of Bias in Systematic Reviews), and Health Evidence [12,13].

Results

Five studies fulfilled the selection criteria and were analyzed in detail. These five articles included one laboratory study, two systematic reviews, one network meta-analysis, and one systematic review and meta-analysis of randomized trials. Two studies concluded that surgical masks could provide equivalent protection to N95 respirators [14,15]. Yin et al. stated that N95 respirators are superior to surgical masks [16]. The fourth study found that better protection can be achieved when the surgical masks are used by the aerosol source (infected person) rather than when the recipient wears the N95 respirator [17]. The last study concluded that surgical masks or N95 respirators alone do not provide complete protection [18]. A summary of the eligible studies is presented in Table 1.

**Table 1:** Summary of the included studies. HCW: health care workers; PPE: personal protective equipment; FFP: filtering facepiece; COVID-19: coronavirus disease 2019; RCT: randomized controlled trial; PICOs: patient/population, intervention, comparison, and outcomes; RPE: respiratory protective equipment

S. No.	Author, year of publication	Title of the paper	Type of study	Summary	Results	Conclusion OR main result
1	Ionescu et al. 2021 [14]	Efficacy of PPE against COVID-19 transmission via dental handpieces	Laboratory study	Patient and operator manikins were used to recreate a dental setting; Suspension with a viral load was injected into the manikin's mouth; The dental procedure was performed with an air turbine handpiece; A quantitative real-time polymerase chain reaction was used to evaluate the effectiveness of surgical masks and N95	When a face shield was not used, virus loads on the exterior surfaces of masks and respirators increased. All respiratory protective equipment had a decrease in viral loads when the shield was worn.	Surgical masks and N95 (FFP2) or FFP3 respirators were equally effective in protecting the operator
2	Bartoszko et al. 2020 [15]	Medical masks vs N95 respirators for preventing COVID-19 in HCWs	Systematic review and meta-analysis of randomized trials	Three electronic databases were searched between January 1, 2014, to March 9, 2020. RCTs comparing the protective efficacy of medical masks to N95 respirators in HCWs were included	A total of 463 references were identified; 12 eligible articles were retrieved for full text; Four RCTs were included for quantitative synthesis.	There is little evidence proofing that N95 respirators and surgical masks provide similar protection against COVID-19
3	Yin et al. 2020 [16]	Comparative efficacy of respiratory PPE against viral respiratory infectious diseases in HCWs	Network meta-analysis	Four electronic databases were searched between January 1, 1970, to December 31, 2019. Studies included were cluster RCTs comparing the effectiveness of respiratory personal protective equipment and wearing manner in preventing healthcare workers from viral respiratory infectious diseases	A total of 745 references were identified; 21 eligible articles were retrieved for the full text; six cluster RCTs were included	The continuous wearing of N95 respirators on the whole shift provides the best protection from viral respiratory infectious diseases
4	de Araujo et al. 2021 [17]	Front lines of the COVID‐19 pandemic: what is the effectiveness of using PPE in health service environments?	Systematic review	Six electronic databases and the grey literature were searched Studies were included or excluded based on the predetermined PICOs	A total of 4820 references were retrieved; 35 articles were selected for a complete reading; 13 articles were included for qualitative synthesis	The hazard of transmission was decreased by using a surgical mask or N95 respirator The use of masks, even those with lower filtration efficiency, by all people in the same area reduces the risk more effectively than the use of high-filtration respirators for just a few people.
5	Samaranayake et al. 2020 [18]	The effectiveness and efficacy of RPE in dentistry and other healthcare settings	Systematic review	Four electronic databases were searched between January 1, 1990, and May 15, 2020. For each database, a single search string was created utilizing (PICOs) search words	A total of 1786 references were retrieved; 310 articles were selected for a complete reading; 20 studies underwent detailed analysis	Surgical masks and N95 respirators when used alone cannot provide absolute protection

Using the AMSTAR-2 tool, all four systematic reviews/meta-analyses in the five assessed articles were classified as critically low-quality reviews. In addition, the four systematic reviews were classified as having a high risk of bias using the ROBIS tool. However, using the Health Evidence tool, the four systematic reviews were assessed; three had strong ratings and one had a moderate rating. A summary of included systematic reviews' risk of bias is shown in Tables 2-4.

**Table 2:** Summary of included studies (systematic reviews/meta-analyses) risk of bias using AMSTAR-2 tool P/Y: partial yes;  No M/A: no meta-analysis; PICOs: patient/population, intervention, comparison, and outcomes; RCTs: randomized control trials; NRSI: non-randomized studies of interventions; AMSTAR: A Measurement Tool to Assess Systematic Reviews

Questions	1. PICOs	2. Protocol	3. Study Design	4. Comprehensive Search	5. Study Selection	6. Data Extraction	7. Excluded Studies Justification	8. Included Studies Details	9. A. Risk of Bias (RCTs)	9. B. Risk of Bias (NRSI)	10. Funding Source	11. A. Meta-analysis results statistical combination (RCTs)	11. B. Meta-analysis results statistical combination (NRSI)	12. Risk of Bias on Meta-Analysis	13. Risk of Bias in Individual Studies	14. Explanation for Heterogeneity	15. Publication Bias	16. Conflict of Interest	Overall
Bartoszko et al. 2020 [15]	Yes	Yes	Yes	P/Y	Yes	Yes	NO	P/Y	P/Y	Only RCTs	No	Yes	No	Yes	No	Yes	No	No	Critically Low-quality reviews
Yin et al. 2020 [16]	Yes	Yes	Yes	P/Y	No	Yes	P/Y	P/Y	P/Y	Only RCTs	No	Yes	No	Yes	No	Yes	No	Yes	Critically Low-quality reviews
de Araujo et al. 2021 [17]	Yes	P/Y	No	P/Y	Yes	Yes	Yes	No	No	No	No	No M/A	No M/A	No M/A	No	No	No M/A	Yes	Critically Low-quality reviews
Samaranayake et al. 2020 [18]	Yes	P/Y	No	P/Y	No	Yes	Yes	P/Y	P/Y	No	No	No M/A	No M/A	No M/A	Yes	No	No M/A	Yes	Critically Low-quality reviews

**Table 3:** Summary of included studies (systematic reviews/meta-analyses) risk of bias using ROBIS tool ROBIS: Risk of Bias in Systematic Reviews

Questions	1. Study eligibility criteria	2. Identification and selection of the studies	3. Data collection and study appraisal	4. Synthesis and findings	Overall
Bartoszko et al. 2020 [15]	Low	High	Low	Low	RISK: High
Yin et al. 2020 [16]	Low	High	Unclear	High	RISK: High
de Araujo et al. 2021 [17]	Low	Low	High	High	RISK: High
Samaranayake et al. 2020 [19]	Low	High	High	High	RISK: High

**Table 4:** Summary of included studies (systematic reviews/meta-analyses) risk of bias using Health Evidence tool PICOs: patient/population, intervention, comparison, and outcomes

Questions	1. PICOs	2. Inclusion criteria	3. Comprehensive search strategy	4. Strategy covers an adequate number of years	5. Described level of evidence	6. Assess method quality	7. Result transparency	8. Combining the findings	9. Method used for combining/comparing the result	10. Funding Source	Overall
Bartoszko et al. 2020 [15]	Yes	Yes	Yes	Yes	Yes	Yes	No	Yes	Yes	Yes	Strong Evidence
Yin et al. 2020 [16]	Yes	No	Yes	Yes	Yes	Yes	Yes	Yes	Yes	Yes	Strong Evidence
de Araujo et al. 2021 [17]	Yes	Yes	Yes	Yes	Yes	No	Yes	Yes	No	No	Moderate Evidence
Samaranayake et al. 2020 [18]	Yes	Yes	Yes	Yes	Yes	Yes	Yes	No	No	Yes	Strong Evidence

Discussion

Surgical Facemasks Versus N95 Respirators

The use of RPE is a powerful protective tool for HCWs [18]. However, several factors can affect their filtration efficiency (e.g. Airflow dynamics, wear time, inhaled particle size, mask wetness, manufacturing quality, and mask fit). [18]. Therefore, according to Samaranayake et al., N95 respirators or surgical masks do not offer complete protection when used individually [18]. In an included laboratory study, patient and operator dummies were used to simulate a dental situation [14]. They found that the outer surfaces of respirators and masks had the greatest virus loads, emphasizing the necessity of removing and disposing of respirators and masks following each patient [14]. They concluded that N95 respirators and surgical masks were both equally effective in protecting the operator safe in a hazardous environment [14]. However, their test was of short duration and could not be deemed for long-lasting procedures [14]. A systematic review concluded that there is no clear evidence that surgical masks are less effective than N95 respirators in protecting HCWs from laboratory-confirmed respiratory viral infections during routine care and non-aerosol-generating procedures [15]. Another systematic review stated that the use of surgical masks by the source of infectious aerosol generators offers higher protection than the use of N95 respirators by the recipient [17]. Accordingly, the risk of exposure can be decreased by having everyone in the area use masks with lower filtration efficiency instead of employing respirators with high filtration efficacy only for some people in the area [17]. Yin et al. found that N95 respirators were superior to surgical masks [16]. They found that wearing N95 respirators continuously throughout a shift can offer better protection against respiratory viral infections [16]. In contrast, the continuous wearing of surgical masks leads to moisture accumulation in the inner layers of the mask, reducing filtration efficiency [16].

Airborne particulate filtering efficacy: Coronavirus infection is transmitted by aerosols and droplets. Accordingly, it is important to evaluate their particle properties and aerodynamic behavior [19]. Frequent sneezing and coughing, or even speaking by COVID-19-infected patients create viral plumes with thousands of droplet sizes varying from 0.6 to 100 μm per cubic centimeter [19, 20]. Under optimal humidity and temperature conditions, aerosol droplets of all sizes can fly up to 7-8 m [19]. Similar to most viruses, the average size of SARS-CoV-2 is about 0.1 μm [19]. With an airflow of 85 l/min, which corresponds to vigorous breathing, the N95 respirators are able to capture 95% of particles with a size of 300 nm [18]. Moreover, N95 respirators offered better protection compared to surgical masks for those particles with less than 20 μm diameter in size thus the efficacy estimates ranged from 2% to 92%.

Wearing time and mask-fit: The protection and prevention efficiency of respiratory protective equipment (RPE) is affected by the wearing time [21]. Compared to surgical masks, continued wearing of N95 respirators during work hours can provide better protection against respiratory infections [16]. Continuous wearing of surgical masks results in moisture accumulation in the inner layer of the mask, leading to a reduced filtration rate and effectiveness [16]. Reports suggest that the reason HCWs contract viral infections when exposed to aerosolized microbes is via leakage from face masks or respirators [18]. If the selected RPE does not provide an adequate seal to the face, even if it has a strong aerosol barrier, its use will not offer the desired protection [17]. The efficiency of RPE is highly dependent on the fit of the RPE worn, for example, the face-fitting competence of N95 respirators is considered a key factor in their preventive effectiveness [18]. In the dental field, wearing a custom-fit N95 respirator with a patent seal all around offers superior protection against infectious bioaerosols [18]. However, prolonged wearing of RPE and other additional PPE compresses the cheeks, forehead, bridge of the nose, and ears, which can be the main reason for head and face pressure and skin damage [22-25]. The lack of proper training in PPE use and successive wearing of them further complicates these issues [21].

Shielding Efficiency of Protective Eyewear and Face Shields

The viral load on the exterior surfaces of masks and respirators is reduced when a face shield is used [14,17]. This marks the effectiveness of face shields in protecting against aerosols and emphasizes the value of using face shields in conjunction with RPE [14]. Protective eyewear and face shields are not only recommended for HCWs but also for people in the risk group [17,26]. A combination of eye protection, face protection, and a properly fitted mask or respirator is required to best protect healthcare workers from respiratory infections and bioaerosols [18]. However, this combination can cause difficulty in breathing, restricted field of vision, headaches, nasal/face pain, and heat stress [21,27,28].

Availability of PPE

The aim of PPE is to control the spread of infection within dental settings. It has always been emphasized that the correct choice of PPE (that provides protection as well as proper fit and comfort) will facilitate compliance with infection control guidelines by dental care providers [27]. However, during the COVID-19 outbreak, dental care providers had to work for hours in full PPE, which led to fatigue and many adverse effects [28]. For instance, headaches, skin irritation, and voice changes were some side effects reported with the prolonged use of masks [28]. In the same context, heat, thirst, bronchospasm, and palpitations were more serious side effects reported with the frequent and prolonged use of PPE [28]. The shortage of PPE (e.g. respirators, masks, gloves, gowns) put patients and medical staff alike at risk during the COVID-19 pandemic [29,30]. At the global level, the demand for N95 respirators was very high, although all kinds of PPE were in demand [30]. HCWs have been increasingly urged to ration and reuse PPE, prompting calls for a government-led reallocation of manufacturing capacity to address mask shortages [31]. Without adequate PPE, the risk to HCWs is increased [32]. 

Systematic Reviews Risk of Bias Assessment Tools

A recent rise has been observed in the number of published systematic reviews and meta-analyses [12,33,34]. This rapid increase in biomedical publications made it almost impossible for healthcare professionals and policymakers to keep up with primary research. Therefore, to provide evidence-based healthcare, healthcare decision-makers greatly rely on systematic reviews. Systematic reviews aim to identify, evaluate and summarize the results of individual studies to make the existing evidence more accessible to decision-makers [13]. However, they can be subjected to a number of biases; hence, it is important to distinguish high-quality from low-quality reviews [34]. Accordingly, many tools have been developed to assess the risk of bias in systematic reviews; however, a few of them are comprehensive. Risk of bias assessment tools come in three types scales, checklists, and items [35]. We used AMSTAR-2 and ROBIS tools as they have been recommended by many studies [12,13,26,30,36-38]. To our knowledge, there are no studies assessing the Health Evidence Tool.

AMSTAR-2: AMSTAR stands for “A MeaSurement Tool to Assess systematic Reviews” [39]. In 2007, AMSTAR was developed and it consisted of 11 items [39]. However, this tool needed some modification to improve it is efficiency [36,40]. Accordingly, AMSTAR-2 was introduced to assist decision-makers in their search for high-quality systematic reviews, particularly ones that are based on non-randomized studies of a healthcare intervention [12]. By concentrating on their methodological quality and expert agreement, the 16 questions in this tool assist in differentiating between the quality of systematic reviews.

ROBIS: Another tool for assessing the risk of bias in systematic reviews is ROBIS ("Risk Of Bias In Systematic reviews") [41]. It consists of three phases; Phase 1 is assessing relevance, which is optional; Phase 2 is identifying concerns with the review process; and Phase 3 is assessing the overall risk of bias [41]. ROBIS demands a more comprehensive evaluation of the systematic review methodology and better comprehension of the addressed clinical subject [37]. Each question on ROBIS included five possible answers, at times, it was difficult to determine the difference between "yes," "probably yes," and "no," or "probably no."

Health Evidence Tool: It is a quality assessment tool designed to appraise systematic reviews and meta-analyses to determine the effectiveness of interventions. This tool includes a total of 10 critical appraisal questions. A total of 10 points indicates the quality rating of the review. A point for each question, 1 point taken if answered "Yes" and 0 if answered "No". To our knowledge, there are no studies assessing the Health Evidence tool. The Health Evidence tool is less sensitive when compared to AMSTAR-2 and ROBIS. Although the Health Evidence tool was easy to use, some questions were not specific. Accordingly, more questions and answer options would improve its sensitivity. The Health Evidence tool can be used as a complementary tool or by those who are not experienced in risk of bias assessment.

So far there is no gold standard for evaluating the quality of systematic reviews [26]. AMSTAR-2 and ROBIS are the most used for risk of bias assessment [13,26,37]. The risk of bias tools has some variation in their theoretical structure, question types, and answer levels [37]. AMSTAR-2 and Health Evidence evaluate the methodological quality of the studies. However, ROBIS places a higher priority on the findings part [37]. According to one study comparing the two tools, AMSTAR-2 had more inconsistent agreements on individual questions compared to ROBIS, which is consistent with our assessment. Also, there are several questions in AMSTAR-2 that, depending on the assessed study results, can evoke varied answers [38]. However, AMSTAR-2 in comparison to ROBIS is simpler and easier to use; using the ROBIS tool took more effort to perform. AMSTAR-2 is a less complex tool, user-friendly, and successful in assessing the quality of systematic reviews including randomized and non-randomized studies. [38]. In general, the three tools can be improved by some modifications, and the decision to apply one or all of these tools should be determined by the researcher's objectives [30]. A summarized comparison between the tools is presented in (Table 5).

**Table 5:** A summarized comparison between AMSTAR-2, ROBIS, and Health Evidence tools. + + + (High), + + ( Moderate), + (Low) PDF: portable document format

The Tool	AMSTAR-2	ROBIS	Health Evidence
Number of Questions	16	21	10
Number of Possible Answers	6	5	3
Available Forms	Online and PDF	PDF	PDF
The Final Result	Auto-generated	Manual	Manual
Sensitivity	+ + +	+ + +	+
Complexity	+ +	+ + +	+
Time Consumption	+ +	+ + +	+

Limitations of the review

There are certain limitations in the current systematic review. First, articles in languages other than English were excluded. This might have led to excluding many relevant articles, keeping in mind that the COVID-19 outbreak started in China and had severe impacts on non-English-speaking countries such as Italy. Therefore, the current findings may have not presented the whole picture. Second, the studies included had a high risk of bias and their methodologies had certain flaws, which in turn might have affected the validity of the results they reached. Third, in the current systematic review, we did not consider the vaccination status, which can have a profound effect on the extent of prevention and as such eliminate the differences between the surgical mask and the N95. Lastly, in the current systematic review, we did not look into the grey literature, which could have added more information to the current findings.

## Conclusions

There is a lack of evidence-based data for a comparative conclusion about the efficacy and superiority of surgical masks vs N95 respirators. Surgical masks and N95 respirators are essential parts of PPE in the post-COVID-19 era. RPE plays a vital role in protecting dentists and other HCWs from respiratory infectious diseases. As good as the protection N95 respirators offer, they can cause several drawbacks. Future studies are needed to compare the efficiency of the different types of N95. According to this systematic review, N95 respirators provided better protection against COVID-19 infection compared to surgical masks. Based on our assessment, AMSTAR-2 is the preferred risk-of-bias assessment tool as it combines both sensitivity and simplicity.
